# Sudden unexpected death in epilepsy and ictal asystole in patients with autoimmune encephalitis: a systematic review

**DOI:** 10.1007/s10072-023-07280-z

**Published:** 2024-01-09

**Authors:** Alberto Vogrig, Fabrizio Bellizzi, Alessandra Burini, Gian Luigi Gigli, Luca Girardi, Jérôme Honnorat, Mariarosaria Valente

**Affiliations:** 1 Clinical Neurology, Department of Medicine (DAME), University of Udine, Udine, Italy; 2 Clinical Neurology, Department of Head-Neck and Neuroscience, Azienda Sanitaria Universitaria Friuli Centrale (ASU FC), Piazzale Santa Maria della Misericordia, 15, 33010 Udine, Italy; 3 Department of Environmental Systems Science, Swiss Federal Institute of Technology (ETH) Zürich, Zurich, Switzerland; 4 French Reference Center for Paraneoplastic Neurological Syndromes and Autoimmune Encephalitis, Hospital for Neurology and Neurosurgery Pierre Wertheimer, Lyon University Hospital, Lyon, France; 5 MeLiS Institute - UCBL-CNRS UMR 5284 - INSERM U1314, Université Claude Bernard Lyon 1, Lyon, France

**Keywords:** Limbic encephalitis, SUDEP, Ictal asystole, Pathophysiology, Autoimmune epilepsy

## Abstract

**Objective:**

As autoimmune encephalitis (AE) often involves the mesial temporal structures which are known to be involved in both sudden unexpected death in epilepsy (SUDEP) and ictal asystole (IA), it may represent a good model to study the physiopathology of these phenomena. Herein, we systematically reviewed the occurrence of SUDEP and IA in AE.

**Methods:**

We searched 4 databases (MEDLINE, Scopus, Embase, and Web of Science) for studies published between database inception and December 20, 2022, according to the PRISMA guidelines. We selected articles reporting cases of definite/probable/possible/near-SUDEP or IA in patients with possible/definite AE, or with histopathological signs of AE.

**Results:**

Of 230 records assessed, we included 11 cases: 7 SUDEP/near-SUDEP and 4 IA. All patients with IA were female. The median age at AE onset was 30 years (range: 15–65), and the median delay between AE onset and SUDEP was 11 months; 0.9 months for IA. All the patients presented new-onset seizures, and 10/11 also manifested psychiatric, cognitive, or amnesic disorders. In patients with SUDEP, 2/7 were antibody-positive (1 anti-LGI1, 1 anti-GABABR); all IA cases were antibody-positive (3 anti-NMDAR, 1 anti-GAD65). Six patients received steroid bolus, 3 intravenous immunoglobulin, and 3 plasmapheresis. A pacemaker was implanted in 3 patients with IA. The 6 survivors improved after treatment.

**Discussion:**

SUDEP and IA can be linked to AE, suggesting a role of the limbic system in their pathogenesis. IA tends to manifest in female patients with temporal lobe seizures early in AE, highlighting the importance of early diagnosis and treatment.

## Introduction

Autoimmune encephalitis (AE) is an emerging neurological disease, whose characteristics and disease mechanisms have been described only recently, and they are far from being completely clarified [1]. AE can be idiopathic or paraneoplastic. The first report of limbic encephalitis associated with cancer was described in 1968 [2]. In the following decades, the presence of autoantibodies targeting intracellular antigens was demonstrated to be an epiphenomenon of the underlying immune response against cancer cells, rather than a direct cause of the neurologic pathology [1]. The recent discovery of neuronal autoantibodies, some of them targeting neuronal surface proteins in patients without cancer, as well as the characterization of precise diagnostic criteria in 2016 [3], contributed to enlighten the frequency and relevance of AE. In a recent study [4], the incidence and prevalence of autoimmune and infectious encephalitis were found to be comparable (respectively, a prevalence of 13.7 vs 11.6/100,000; incidence of 0.8 vs 1.0/100,000 person/years). Moreover, this study showed a rise in the incidence of AE over the years (from 0.4 in the decade 1995-2005, to 1.2 in the decade 2005-2015), probably related to improved diagnostic techniques.

AE is a heterogeneous disease. Its clinical features include impairment of working memory and cognition, psychiatric symptoms, altered sensorium, gait instability, movement disorders, dysautonomia, and seizures. The clinical presentation can be challenging, because of the subacute course of the disease, and the wide number of autoantibodies implied. There are no pathognomonic symptoms: the diagnosis is based on clinical, paraclinical, laboratory, and imaging criteria combined [3]. Nevertheless, seizures and electroencephalogram (EEG) abnormalities are important features of AE and are very common even at disease onset [1, 5].

Epilepsy can be associated both with sudden unexpected death in epilepsy (SUDEP) and ictal asystole (IA). SUDEP is defined as “sudden, unexpected, witnessed or unwitnessed, nontraumatic and non-drowning death, occurring in benign circumstances, in an individual with epilepsy, with or without evidence for a seizure and excluding documented status epilepticus (SE), in which postmortem examination does not reveal a cause of death” [6]. The incidence of SUDEP, according to a recent meta-analysis, is 0.8 to 1.2 cases per 1000 people with epilepsy per year [7]. The pathophysiology of SUDEP is not completely understood. Current knowledge shows a higher risk in patients with non-controlled, generalized, tonic-clonic seizures (GTCS), and in adults aged 18 to 35 years compared to children [7].

IA is defined as an R-R interval of 3 s or more during an ictal event [8]. It can be differentiated in “not very prolonged” (< 30 s of duration) and “very prolonged” (> 30 s). In contrast with SUDEP, IA is associated with focal epilepsy, even if it can occur after bilateral tonic-clonic evolution in a minority of cases, especially with very prolonged seizures. IA does not seem to be associated with a fatal course or a higher risk of SUDEP [9]. However, the implantation of a pacemaker may be necessary since IA is associated with a higher risk of injuries and/or falls when epilepsy cannot be controlled adequately by antiseizure medications (ASMs) or surgery [9].

As AE often involves the mesial temporal structures which are known to be possibly involved in both SUDEP and IA [10], it may represent a good model to study the physiopathology of these phenomena. Therefore, to shed light on their mechanisms, we systematically reviewed the existing literature on SUDEP and IA cases in patients with AE.

## Methods

We systematically reviewed the existing literature searching 4 databases (MEDLINE, Scopus, Embase, and Web of Science) for studies published between database inception and December 20, 2022, according to the Preferred Reporting Items for Systematic Reviews and Meta-Analyses (PRISMA) [11]. For the literature search, 2 groups of keywords were selected, the first one to identify SUDEP or IA cases, and the second one to find AE cases (Table 1). Each term of the first group was combined with each term of the second one. To make the search more efficient (i.e., to avoid duplicate results from the same database), a single-entry line for each database was written by an engineer (L.G.) using a code on MatLab with the search terms provided.

**Table 1:** Keywords used for database search

Keywords group 1 (SUDEP or IA)	Keywords group 2 (AE)
SUDEP	Limbic
Sudden unexpected death in epilepsy	Autoimmune epilepsy
Ictal asystole	Encephalitis
Ictal central apnea	Neur* antibod*
	Anti-Hu
	Ma2
	GABAB receptor
	GABABR
	GABAB-R
	AMPA receptor
	AMPAR
	AMPA-R
	LGI1
	LGI-1
	GAD
	GAD65
	GAD-65
	NMDA receptor
	NMDAR
	NMDA-R

Two groups of keywords were selected, the first one to identify sudden unexpected death in epilepsy (SUDEP) or ictal asystole (IA) cases, and the second one to find autoimmune encephalitis (AE) cases. We combined each term of the first group with each term of the second one, writing a single-entry line for each database

In Scopus, the search was limited within article title, abstract, and keywords. No filters were applied in the remaining databases. We included all cases found in the literature of definite SUDEP, definite SUDEP plus, probable SUDEP, probable SUDEP plus, possible SUDEP, near-SUDEP, and near-SUDEP plus as defined by Nashef et al. 2012 [6] or IA according to van der Lende et al. 2015 [8], in patients with possible AE/definite autoimmune limbic encephalitis as defined by Graus et al. 2016 [3], or with histopathological signs of limbic AE. A summary of the definitions adopted is provided in Table 2. No age limit was used. Cited reference searching was also conducted.

**Table 2:** Definitions of SUDEP, ictal asystole, and autoimmune encephalitis

Term	Definition
Definite SUDEP	Sudden, unexpected, witnessed or unwitnessed, nontraumatic and nondrowning death, occurring in benign circumstances, in an individual with epilepsy, with or without evidence for a seizure and excluding documented status epilepticus (seizure duration < 30 min or seizures without recovery in between), in which postmortem examination does not reveal a cause of death
Definite SUDEP plus	Satisfying the definition of definite SUDEP, if a concomitant condition other than epilepsy is identified before or after death, if the death may have been due to the combined effect of both conditions, and if autopsy or direct observations/recordings of terminal event did not prove the concomitant condition to be the cause of death
Probable SUDEP/probable SUDEP plus	Same as definite SUDEP but without autopsy. The victim should have died unexpectedly while in a reasonable state of health, during normal activities, and in benign circumstances, without a known structural cause of death
Possible SUDEP	A competing cause of death is present
Near-SUDEP/near SUDEP plus	A patient with epilepsy survives resuscitation for more than 1 h after a cardiorespiratory arrest that has no structural cause identified after investigation
Ictal asystole	An R-R interval of 3 s or more at the EEG during an ictal epileptic event
Definite autoimmune limbic encephalitis	All four* of the following criteria: 1. Subacute onset (rapid progression of less than 3 months) of working memory deficits, seizures, or psychiatric symptoms suggesting involvement of the limbic system 2. Bilateral brain abnormalities on T2-weighted fluid-attenuated inversion recovery MRI highly restricted to the medial temporal lobes† 3. At least one of the following: - CSF pleocytosis (white blood cell count of more than five cells per mm^3^) - EEG with epileptic or slow-wave activity involving the temporal lobes 4. Reasonable exclusion of alternative causes *If one of the first three criteria is not met, a diagnosis of definite limbic encephalitis can be made only with the detection of antibodies against cell-surface, synaptic, or onconeural proteins †18 Fluorodeoxyglucose (18F-FDG) PET can be used to fulfill this criterion. Results from studies from the past 5 years suggest that 18F-FDG-PET imaging might be more sensitive than MRI to show an increase in FDG uptake in normal-appearing medial temporal lobes
Possible autoimmune encephalitis	All three of the following criteria: 1. Subacute onset (rapid progression of less than 3 months) of working memory deficits (short-term memory loss), altered mental status*, or psychiatric symptoms 2. At least one of the following: - New focal CNS findings - Seizures not explained by a previously known seizure disorder - CSF pleocytosis (white blood cell count of more than five cells per mm^3^) - MRI features suggestive of encephalitis† 3. Reasonable exclusion of alternative causes *Altered mental status defined as decreased or altered level of consciousness, lethargy, or personality change †Brain MRI hyperintense signal on T2-weighted fluid-attenuated inversion recovery sequences highly restricted to one or both medial temporal lobes (limbic encephalitis), or in multifocal areas involving grey matter, white matter, or both compatible with demyelination or inflammation

The definitions adopted in the present study are reported herein, using previous diagnostic criteria [3, 6, 8]

**Fig. 1:**
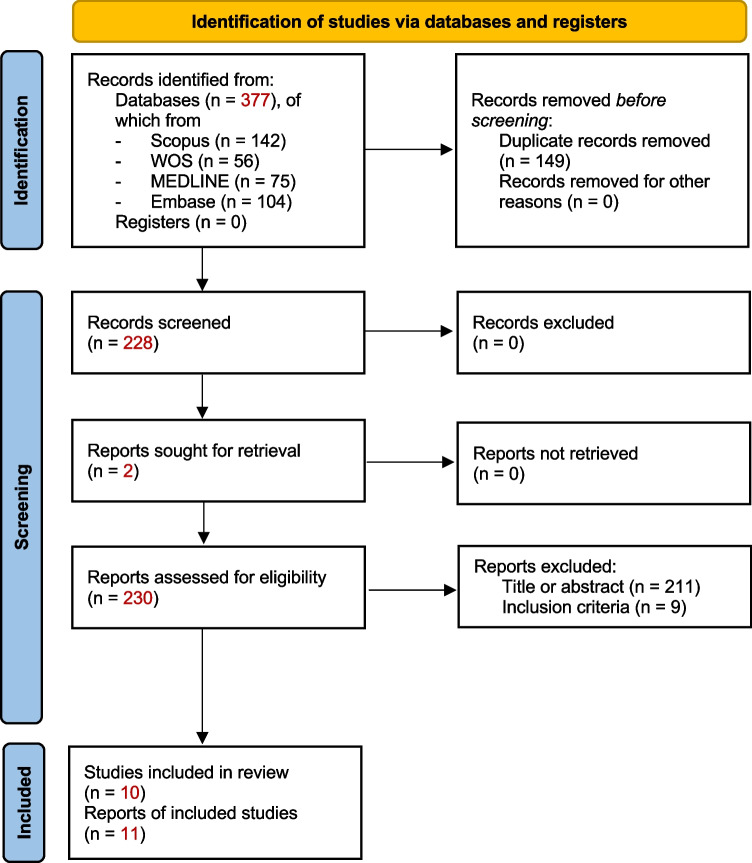
PRISMA flow diagram. The systematic review process is summarized here. From: Page MJ, McKenzie JE, Bossuyt PM, Boutron I, Hoffmann TC, Mulrow CD, et al. The PRISMA 2020 statement: an updated guideline for reporting systematic reviews. BMJ 2021;372:n71. https://doi.org/10.1136/bmj.n71

Two medical doctors (A.B. and F.B.) screened each record to establish concordance with the inclusion criteria (Fig. 1). No automation tool was used. When a disagreement was found, a neurologist with expertise in both AE and epilepsy (A.V.) provided final decision on whether to include a study.

## Results

### Demographic and event-related data

Of 230 records assessed, a total of 10 studies with 11 individual cases of AE with SUDEP (*n* = 7) or IA (*n* = 4) were included (Table 3) [12–21]. Two patients were male and 8 females; in 1 case, the sex of the patient was not specified. Considering the whole cohort, the mean age at the event was 34.9 ± 15.9 years (median 30). Among patients with SUDEP, the mean age at the event was 41.0 ± 15.3 years. In the IA group, the mean age at the event was 24.3 ± 11.5 years.

**Table 3:** Summary of the included cases

Pt	Paper (first author)	Sex	Age at the event (yrs)	Onset-event delay (mo)	Event: SUDEP or IA	AE diagnosis	Type of seizure (if any) during which the event happened	Types of seizure before the event	Seizure semiology	Frequency of seizures before the event (n/month)	AE and epilepsy therapy	MRI	CSF	Ab	Follow-up
1	Hebel	UN	49	3	Definite SUDEP	Histopathological signs of inflammation in the R hippocampus and amygdala	Nocturnal focal to bilateral tonic–clonic seizure	Not known	Head version to the L, then GTCS	0.3	None	R mesial temporal alterations	Not known	Not found	NA
2	Rizzi	M	55	6	Definite SUDEP plus	Possible AE	GTCS	Focal and GTCS	(1) R hand paresthesia and automatisms, R limbs contraction, mouth deviation, and possible i.a. and falling; (2) FBDS; (3) screaming, neck extension, clonic contractions of the limbs, apnea, and cardiac arrest	120	LEV + OXC, LOR, CLB	L amygdala and hippocampus alterations	Pathological (OCB)	LGI1	NA
3	Ramanathan	F	27	43	Probable SUDEP	Possible AE	Not known	Focal seizures, NCSE	R facial twitching, R limbs jerking, NCSE	4	Steroid bolus, oral steroid, LEV, TPM, CLB	Normal	Not known	Not found	NA
4	Ramanathan	F	26	42	Probable SUDEP	Possible AE	Not known	Focal seizures, NCSE	Head and eyes deviation to the L, L hemi-paresthesia, L facial and upper limb jerking, NCSE	2	Steroid bolus, CBZ, VPA, TPM	R temporal alterations	Pathological (OCB)	Not found	NA
5	Ovens	M	65	0	Near SUDEP	Possible AE	Not known	GTCSs after near-SUDEP event	GTCS	0	IVIg, CBZ, etoposide, carboplatinum	Normal	Pathological (OCB)	GABABR, (Hu)	Improved (only mild memory deficits) at 1 year
6	Afshari	F	31	11	Definite SUDEP	Possible AE (Hashimoto's encephalopaty)	Not known (found dead in her bed)	Focal seizures, secondarily GTCSs	(1) Staring, hand and oral automatisms; (2) staring, head version to the L or R side, GTCS	2	Steroid bolus, plasmapheresis, PHT, VPA, LTG, LEV; at the end, PB and CBZ	Normal	Pathological	Not found	NA
7	Inayat	F	34	12	Near SUDEP	Probable anti-NMDAR AE	GTCS	No previous seizures	GTCS (previous episodes of intermittent unresponsiveness; facial twitching)	0	IVIg, plasmapheresis, LCM; tumor resection	Not specified	Normal	Not found	Improvement with return to ADL and to work at 1.5 months
8	D'Souza	F	22	24	Two IAs	Definite AE	Focal seizure (secondarily generalized?)	Focal seizures with i.a	Dizziness, metallic smell, déjà-vu, i.a., R or L hand flexion, lower limbs stiffening	1	BRV and LTG	Normal	Normal except for GAD65 Antibodies	GAD65	5 seizures occurring monthly at 6 months
9	Lee	F	41	0	IA	Definite anti-NMDAR AE	Focal seizure	Focal seizures with i.a	Focal seizures with i.a	Not known ("frequent")	Steroid bolus, oral steroid, TPM, LEV; PM implantation	L mesial temporal lesion	Pathological (OCB)	Anti-NMDAR	Only minor anterograde memory impairment
10	Ziaeian	F	19	1	IA	Definite anti-NMDAR AE	Not known (focal SE?)	GTCS, focal SE	(1) GTCS; (2) NCSE with emotional lability, cognitive difficulties, and intermittent music hearing	8	Steroid bolus, oral steroid, plasmapheresis, LEV; temporary PM; tumor resection	Normal	Pathological	Anti-NMDAR	Improvement of cognitive and memory abilities at 5 months
11	Millichap	F	15	0,87	Multiple IAs	Definite anti-NMDAR AE	Focal seizures with L-temporal onset and secondary generalization	Focal seizures	Cutaneous sensory aura, staring, confusion, raising of the arms, sometimes with bilateral tonic extension of the 4 limbs	≥ 4	Steroid bolus, oral steroid, IVIg, PB, then FOS and LEV; PM implantation; tumor resection	L posterior temporal and mesial temporal alterations	Pathological	Anti-NMDAR	Only subtle working memory deficits at 4 months

We included 11 patients with autoimmune encephalitis and SUDEP or ictal asystole in the present review. Their clinical, imaging, and laboratory data are summarized here

*Abbreviations*: *ADL* activities of daily living, *AE* autoimmune encephalitis, *CBZ* carbamazepine, *CLB* clobazam, *FBDS* faciobrachial dystonic seizures, *FOS* fosphenytoin, *GTCS* generalized tonic–clonic seizures, *IA* ictal asystole, *i.a.* impaired awareness, *IVIg* intravenous immunoglobulin, *L* left, *LCM* lacosamide, *LEV* levetiracetam, *LOR* lorazepam, *LTG* lamotrigine, *NA* not available, *OCB* oligoclonal bands, *OXC* oxcarbazepine, *PB* phenobarbital, *PHT* phenytoin, *PM* pacemaker, *R* right, *SE* status epilepticus, *SUDEP* sudden unexpected death in epilepsy, *TPM* topiramate, *VPA* valproic acid

The comorbidities of patients were as follows: hypertension for 2 patients (both with SUDEP); 1 SUDEP patient had mild depression; 1 was a smoker. One patient (IA) suffered from migraine headaches. During hospitalization, 2 patients with anti-NMDAR encephalitis (and IA) were found to have ovarian teratoma, while 1 patient with SUDEP was found to have metastatic small cells neuroendocrine lung tumor. Of note, not all papers described in detail the comorbidities of patients. The mean delay between the onset of AE symptoms and the event (SUDEP or IA) was 13.0 ± 16.3 months (median 11). For the SUDEP group, it was 16.7 ± 18.1 months (median 11) and for the IA group 6.5 ± 11.7 months (median 0.9). All patients with IA were antibody-positive (3 anti-NMDAR, 1 anti-GAD65).

Regarding patients with SUDEP, 2 had definite SUDEP, 2 probable SUDEP, 2 near SUDEP, and 1 definite SUDEP plus. This latter experienced a cardiac arrest and prolonged apnea during a seizure and the pathological examination found myocardial infarction with no coronary pathology. Among the 2 patients who had near SUDEP, 1 had bradycardia (5 s sinus-pauses) and asystolic cardiac arrest requiring 4 min of cardiopulmonary resuscitation; the other one had ictal *torsade de pointes* and cardiac arrest after a GTCS, treated with epinephrine and defibrillation with a return of spontaneous circulation after 2 defibrillation attempts.

Regarding patients with IA, the mean duration of the event was 17.6 ± 7.4 s. These events should be labelled as “not very prolonged IA”, as the longest episode lasted 28 s, and the shortest 9 s. Among patients with SUDEP, the type of seizures before the event (when described) was focal for 4 patients out of 7; 2 patients had generalized tonic–clonic seizures before the event, 1 patient had both focal and/or GTCS. The SUDEP event occurred in concomitance with GTCS in 3 patients, but for the other 4 patients the description of the seizure type was not present.

Among patients with IA, the type of seizure before the event was focal in 3 out of 4. Only 1 patient had GTCS and focal SE.

### Clinical features of AE

All patients had newly onset seizures; 10 of 11 patients had also psychiatric or cognitive symptoms, varying from memory impairment to depression, agitation, mania, and aggressivity.

A lumbar puncture was performed in all patients, with a pathological cerebrospinal fluid (CSF) analysis in 8 of 11 (4 from the SUDEP group, and 4 from the IA group). For 2 patients (with SUDEP), CSF data were not specified in 1 case or partially specified in the other (only absence of oligoclonal bands and of pleocytosis). For 1 patient with SUDEP-plus, CSF was pathological only for the presence of oligoclonal bands. Five patients had pleocytosis, with a range of 10 to 134 cells/μL. Protein content was normal for 5 patients. Three patients (2 with IA and anti-NMDAR encephalitis, 1 with SUDEP) had increased CSF proteins. CSF glucose level was altered in 2 patients (1 with SUDEP and 1 with IA). For the other patients, it was normal (*n* = 6), or not specified (*n* = 3). Oligoclonal bands were found in 4 patients. For the others, they were absent (*n* = 2, of which 1 with IA and 1 with SUDEP) or not specified (*n* = 5). The PCR for neurotropic viruses was negative (*n* = 4) or not specified (*n* = 7).

A brain MRI was performed in all patients, which was normal in 5 of 11 patients. For the other 6 patients, alterations were found in the right mesial temporal structures (2 SUDEP patients), left mesial temporal structures (1 patient with SUDEP and 1 with IA), left posterior temporal lobe cortex and mesial temporal lobes (1 patient with IA). For 1 patient with SUDEP, brain MRI only showed the signs of chronic cerebrovascular disease. For 2 patients of the 5 with no MRI alterations, brain PET was performed, showing bilateral hypometabolism on the temporal regions for 1 of them (who had IA) and on both inferior frontal and temporal regions for the other one (a SUDEP case).

In the SUDEP group, in 1 patient AE was diagnosed only at postmortem examination, which showed signs of inflammation and lymphocyte infiltration in the right amygdala and hippocampus. Five patients out of 7 fulfilled the criteria for possible AE and 1 patient had probable anti-NMDAR encephalitis. All IA patients had definite AE. Three out of 4 had anti-NMDAR encephalitis; the fourth one had anti-GAD antibodies. Only 2 of 7 SUDEP patients had antibodies in the serum or CSF (1 anti-LGI1, 1 anti-GABABR).

### EEG

An EEG was performed in all patients. When reported, seizure onset was always fronto-temporal or temporal, sometimes bilateral, and the interictal EEG showed diffuse (*n* = 3) or localized slowing restricted to the fronto-temporal regions (*n* = 1) or, in a single case, fronto-temporal spikes that diffused during sleep.

### Treatment

Seven patients were treated with ASMs before the event and 3 were not; for 1 patient, data on ASMs were not provided. Among patients under ASM treatment, 4 had SUDEP and 3 IA. Among patients without ASM treatment, all 3 had SUDEP. At the end of follow-up, all patients received ASMs, except for the one who died after his second seizure (the first seizure was not treated). Nonetheless, some patients started ASMs only after the event (IA or near SUDEP).

Regarding immunotherapy, 6 patients out of 11 were treated with steroid bolus and 5 of them received also oral steroids. Intravenous immunoglobulin were used for 3 patients, 1 with IA and 2 with SUDEP. Three patients (including the one who died after the second seizure) did not receive any immunotherapy. Other treatments included pacemaker implantation for 3 patients with IA (but it was proposed for 4 patients); plasmapheresis for 2 patients with SUDEP and 1 patient with IA; and the resection of teratoma for 3 patients with anti-NMDAR encephalitis, 2 with IA and 1 with near-SUDEP. Carboplatin and etoposide were given to the patient with metastatic small cells lung cancer.

### Follow-up

All the 6 patients who survived the event (2 near SUDEP and 4 IA) improved their symptoms and seizure control at last follow-up, except for 1 (IA patient with anti-GAD encephalitis), who continued to suffer a mean of 5 seizures monthly (Table 3). The efficacy of immunotherapy, along with the resection of tumor (ovarian teratoma), was remarkable for all patients who survived the events.

## Discussion

Autonomic manifestations of seizures are very common in some patients with epilepsy, especially in those with temporal lobe epilepsy (TLE). These can range from goosebumps, epigastric sensations, palpitations, syncope, arrhythmias, heat or cold sensations, sexual arousal, respiratory changes [22].

Ictal asystole also occurs more often in TLE. Importantly, ictal asystole and bradycardia are associated with an increased risk of injuries and traumatic falls. Mechanisms that lead to IA are not fully understood. IA can also lead to syncope, especially if asystole lasts more than 8 s (loss of consciousness and atonia are very common in these cases) [22]. Patients suffering from IA were previously considered at higher risk of SUDEP, but this link has been challenged by multiple studies [9]. The phenomenon of IA can be explained by a parasympathetic outflow or a vasovagal reflex. Asystole leads to cerebral anoxia, which in turns contributes to seizure cessation. This is the reason why IA is considered as a relatively benign phenomenon [22]. The necessity of pacemaker implantation is a matter of debate and may be considered for patients at high risk of falls because of IA-related syncope [9]. It is already known that people with epilepsy have a 2 to 3 times higher risk of death than the general population [23]. SUDEP is the most common major cause of death in people with epilepsy, along with accidents and SE. Nevertheless, the pathogenesis of SUDEP has not yet been completely understood.

**Fig. 2:**
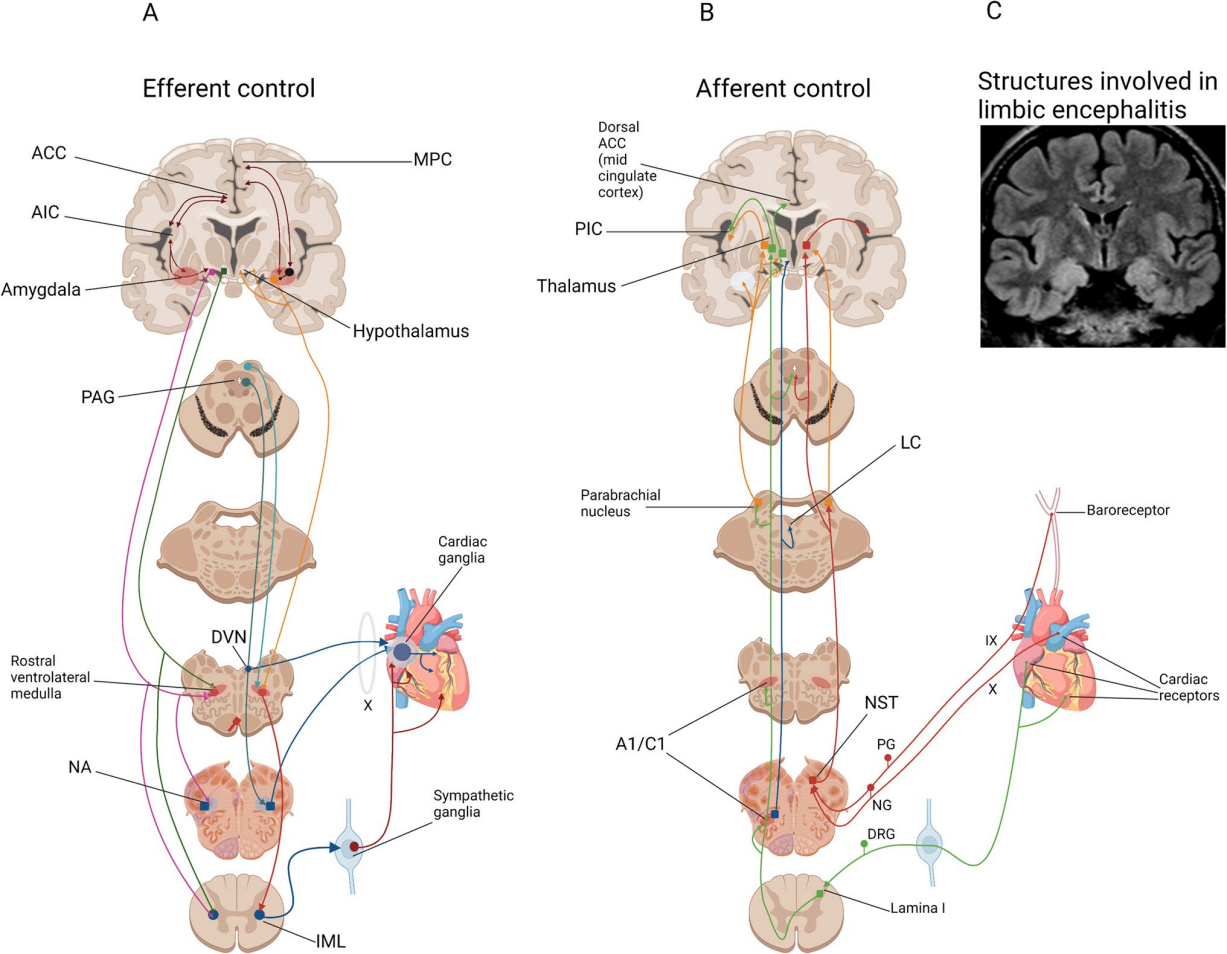
Central control of the heart and potential roles of brain structures affected in autoimmune encephalitis. Brain areas that contribute to the heart function control include the anterior cingulate cortex (ACC), central nucleus of the amygdala, and several hypothalamic nuclei. They act through the medullary and spinal nuclei (efferent control, left). The neurons in the rostral ventrolateral medulla project to the preganglionic sympathetic neurons of the intermediolateral (IML) cell columns in spinal cord and ultimately produce sympathetic activation of the cardiac plexus (paravertebral ganglia). The parasympathetic control derives from the ventrolateral portion of nucleus ambiguus (NA) and the dorsal motor nucleus of the vagus nerve. On the afferent side, spinal neurons activate neurons of the lamina I, which project to the thalamus, parabrachial nucleus (PN), Periaqueductal grey (PAG), and other structures of the hypothalamus and brainstem. The nucleus of the solitary tract (NST) receives inputs from the cardiac vagal neurons and carotid baroreceptors. The thalamic relay nuclei that receive cardiovascular inputs project to the posterior insular cortex. The hypothalamus, PAG, and locus coeruleus (LC) receive viscerosensory afferent fibers from the A1/C1 neurons of the ventrolateral medulla. AIC: Anterior Insular Cortex; DRG: Dorsal root ganglia; DVN: dorsal vagal nucleus; MPC: Medial Prefrontal Cortex; NG: Nodose ganglion; PIC: Posterior Insular Cortex; PG: Petrosal ganglion. Figure created with BioRender.com and adapted from [24] and review of the literature. A typical brain magnetic resonance imaging (coronal section) of a patient with autoimmune encephalitis with temporal seizures is shown (right), demonstrating mesial temporal lobes hypersignal. The patient had anti-LGI1 encephalitis

The central autonomic network (CAN) is estimated as the main actor of ictal autonomic alterations [22, 24]. It is composed of various brain areas, including amygdala, anterior cingulate, insula, thalamus, hypothalamus, periaqueductal grey matter, parabrachial nucleus, and several medullary regions. CAN is important in the homeostasis, conscious visceral perception, and regulation of emotional responses via the sympathetic and parasympathetic systems. A schematic representation of CAN is shown in the Fig. 2, based on the work made by Palma and Benarroch [24]. The core of the CAN is composed by the left amygdala, right and left insulae, and midcingulate cortices. Seizures can activate the CAN through multiple pathways: direct activation, reflex responses to the effects of the seizures, as well as catecholamines release by the adrenal glands. Neurohumoral factors are also implicated, and some evidence show that chronic epilepsy affects hypothalamic and pituitary-adrenal axis. Indeed, the neurohumoral response to stress is higher in people with TLE than healthy controls [25]. Other studies have shown brainstem atrophy in people with SUDEP, suggesting a potential role of autonomic disfunction of respiratory/vagal centres in these patients [26].

The semiology of seizures in LE depends on the involvement of the mesial temporal structures. It is well described in the literature and includes visceral-sensory, autonomic, and emotional auras (epigastric sensations, nausea, alterations in cardiac or respiratory functions, piloerection, flushing, pallor, vomiting, mydriasis, sensation of fear, sadness, pleasure, or anxiety) usually followed by impaired awareness, staring, oral or gestural automatisms (typically ipsilateral to the epileptogenic zone), ictal speech (usually with right-sided seizure foci) or aphasia (mostly with left-sided foci), verbal automatisms, unilateral dystonic posturing (contralateral to the epileptogenic zone), and versive head deviation (contralateral to the epileptogenic zone if occurring just before bilateral spreading). Finally, in the postictal phase, confusion, aphasia, and amnesia for the event can be reported. Of note, ictal apnea and tachycardia are often related to the involvement of the amygdala [27–32]. Moreover, anti-LGI1 encephalitis is associated with bradyarrhythmias [33, 34], which may occur even in the interictal periods. Zhao-Fleming et al. [33] studied this phenomenon by examining LGI1 expression in the cardiac tissues of human donors and mice, finding that murine and human cardiac tissues express LGI1 (mRNA and protein). Therefore, they proposed that autoantibodies may elicit arrhythmias by a direct effect on cardiomyocites [33].

AEs, which is known to involve most the neuronal circuits described above [10], are associated with increased mortality and frequent autonomic symptoms, including IA. A retrospective observational study on 100 patients described a global mortality rate of 15% for anti-NMDAR, anti-LGI1, and anti-GABABR encephalitis together. A later age at onset, admission to intensive care unit (ICU), SE, and anti-GABABR encephalitis were found to be associated with a higher risk of death [35]. More specifically, a higher mortality is described for anti-GABABR encephalitis (41.7%), followed by anti-NMDAR encephalitis (4–10%), and anti-LGI1 encephalitis (2.8%) [35–37]. Also, predictors of death for anti-NMDAR encephalitis are coma at admission (Glasgow coma scale ≤ 8), a higher number of complications, and admission to ICU [36]. Most adult patients with anti-NMDAR need ICU admission (50–69%), and 61% of them have dysautonomia [38]. The most frequently reported causes of death for anti-NMDAR encephalitis are multi-organ failure, pneumonia, and refractory SE [36].

In our systematic review, 3 out of 4 patients who experienced IA had anti-NMDAR encephalitis, and they all had the event within the first month from AE onset. Dysautonomia tends to manifest early in the disease course of these patients and physicians need to be prepared to treat it. Finally, all patients with IA were female, confirming an association already highlighted in the literature: a review by Tényi et al. [9] on IA cases showed a female predominance in new-onset IA. This could be explained by a grater parasympathetic tone in females than in males. In fact, hormones like estrogens, oxytocin and prolactin can increase the parasympathetic tone [9, 39]. Moreover, an ovarian teratoma is a triggering factor for paraneoplastic anti-NMDAR encephalitis [40], and 2 patients from our review had IA and anti-NMDAR encephalitis with ovarian teratoma.

In the reported cohort of SUDEP and IA cases, the patients were young and demonstrated typical clinical, neuroimaging, and neurophysiological features of AE. SUDEP occur a median 11 months from AE onset and therefore appear to be a preventable event, using ASMs and immunotherapy. Immunotherapy is an effective, disease-modifying therapy and can be even more incisive on seizure control than ASMs. Five out of 6 patients who survived the event (IA or near SUDEP) improved their clinical status in the following months; only 1 patient with anti-GAD65 encephalitis had a poor seizure outcome, in line with what we know from the literature [41].

We would like to underline how the definition of near-SUDEP is arbitrary, as IA can be turned into near-SUDEP if cardiopulmonary resuscitation is carried out during the episode, as previously highlighted in the literature [42].

## Conclusions

IA and SUDEP are important risks for people with epilepsy, and their pathophysiology is only partially understood. However, the involvement of autonomic pathways is a major hypothesis. TLE is associated with autonomic manifestations during or after seizures. Therefore, people with limbic AE can be at higher risk for these events. The results of the present study show that people with limbic AE can manifest SUDEP and IA along with other autonomic alterations.

Our review is limited by the few cases we found in the literature, which do not allow us to conclude for a major risk of SUDEP/IA in AE patients. Nevertheless, we hope that this work can emphasize the possible link between autonomic alterations in AE-related TLE and SUDEP or IA, promoting the research and stimulating clinicians to describe these cases in literature.

The presence of a link between these pathologies can have extremely important implications: AE is a treatable cause of epilepsy, so treating these patients can lead to a diminished risk of death (because of SUDEP) and of falls and injuries (because of asystole and syncope).

## Data Availability

The authors confirm that the data supporting the findings of this study are available within the article.
